# Insights into the skeletal muscle characteristics of three southern African antelope species

**DOI:** 10.1242/bio.20149241

**Published:** 2014-10-17

**Authors:** Tertius Abraham Kohn

**Affiliations:** UCT/MRC Research Unit for Exercise Science and Sports Medicine, Department of Human Biology, PO Box 115, Newlands 7725, South Africa

**Keywords:** Wild animals, Enzymes, Cross-sectional area, Fibre type, Metabolism

## Abstract

Skeletal muscle fibre type, cross-sectional area (CSA), maximum enzyme capacities and fibre oxidative capacities were investigated in three southern African antelope species. Muscle samples from blesbok (*Damaliscus pygargus phillipsi*), mountain reedbuck (*Redunca fulvorufula*) and greater kudu (*Tragelaphus strepsiceros*) were collected *post mortem* from the *Vastus lateralis* and analysed for myosin heavy chain (MHC) isoform content, citrate synthase (CS), 3-hydroxyacyl Co A dehydrogenase (3-HAD), phosphofructokinase (PFK), lactate dehydrogenase (LDH) and creatine kinase (CK) activities. Histochemistry and immunohistochemistry were performed to determine relative fibre oxidative capacity, fibre type and cross-sectional area (CSA). Type IIX fibres were the most abundant fibre type in all three species, ranging from 43 to 57%. Kudu had less type IIX fibres than mountain reedbuck and blesbok (*P*<0.05), values confirmed by their respective MHC isoform content. Blesbok had the smallest fibres, followed by mountain reedbuck and finally kudu (*P*<0.001). Overall, all three species had high oxidative and glycolytic capacities, but species differences were found. Kudu had the lowest CS activity, followed by blesbok and mountain reedbuck, but the highest PFK, LDH and CK activities. This study confirmed large variation in oxidative capacities within a single fibre type, as well as overlap between the fibre types with no distinct differences between the three species. The fibre type profile of each species is discussed and confirms some of their physical attributes and capabilities.

## INTRODUCTION

There are more than 5 000 wild mammalian species in the world, yet very little is known about their skeletal muscle properties and function. Most of the current data on skeletal muscle are derived from the study of domesticated animals (rodents, cats, dogs, pigs, horses), frogs and humans. These comparative studies have greatly advanced our understanding of muscle function with regards to strength, endurance capacity and overall sporting performance. However, data on those species that are the fastest, strongest and have a natural capacity for endurance are very limited. Studying these natural occurring athletes (wild animals) can assist us to better understand muscle physiology in the context of genetics, metabolism and structure.

The southern part of Africa is host to a number of phenomenal wild animal species, either possessing the ability to sprint at great speeds (e.g. cheetah and lion) or being able to withstand fatigue for long periods of time, or possessing both of these attributes (e.g. wildebeest and springbok) (Curry et al., 2012; Kohn et al., 2011a; Kohn et al., 2011b; Williams et al., 1997). There is no doubt that these wild animals are the pinnacle of sporting performance, being unaffected by genetic interference (e.g. breeding for specific characteristics) from human manipulation, and subjected to the principle of survival of the fittest. However, their underlying performance ability and capacity are poorly understood, especially on skeletal muscle level.

Muscles from large mammals generally contain three fibre types, namely type I, IIA and IIX, arising from the myosin heavy chain (MHC) isoform each expresses (Bottinelli, 2001). Each isoform differs in their respective hydrolysis rate of ATP, determined primarily by the different activities of the ATPase enzyme embedded in the MHC (Bottinelli et al., 1994). The result is fibres that differ in contractile properties (slow *vs.* fast twitch) and hence will prefer different fuel sources (e.g. fat *vs.* glycogen) (Essén-Gustavsson and Henriksson, 1984). Therefore, the unique functional properties of a muscle group (e.g. gastrocnemius *vs.* soleus) arise from different fibre type combinations, which is primarily determined by the innervating motor neuron (Pette, 1985). Historically, pure type I (slow oxidative) fibres are slow in contraction speed, expresses only MHC I, contain large numbers of mitochondria and are known to be fatigue resistant (Bottinelli, 2001; Schiaffino and Reggiani, 1996). In order to produce the required ATP for contraction, they are able to efficiently metabolise fat, glucose and glycogen aerobically, by having high activities of citrate synthase (CS), 3-hydroxyacyl Co A dehydrogenase (3HAD), but low activities of phosphofructokinase (PFK), lactate dehydrogenase (LDH) and creatine kinase (CK) (Essén-Gustavsson and Henriksson, 1984; Kohn et al., 2007b; Pette, 1985). On the other hand, pure type IIX fibres (fast glycolytic) express only the MHC IIx isoform, giving rise to a fibre that can contract very fast compared to type I fibres (Bottinelli, 2001). As they contain very few mitochondria (low CS and 3HAD activities), their capacity to produce ATP from anaerobic metabolism of glucose, glycogen and phosphocreatine stores is high, reflected by high activities of LDH, PFK and CK. Consequently, this fibre type fatigues quickly due to limited fuel storage capacity. Type IIA fast oxidative fibres, expressing MHC IIa, are slightly slower in contraction speed than type IIX fibres, but contain large numbers of mitochondria and produce ATP from both aerobic and anaerobic metabolism, rendering this fibre type more resistant to fatigue (Kohn et al., 2007b; Pette, 1985; Schiaffino and Reggiani, 1996). The type IIB fibre type (derived from expressing MHC IIb) is abundant in rodent limb muscles, and only trace amounts have been found in cheetah, llama and pig limb muscles (Graziotti et al., 2001; Hyatt et al., 2010; Kohn and Myburgh, 2007; Toniolo et al., 2004). Thus far, most of the larger mammalian species investigated had no expression of the MHC IIb isoform in their limb muscles, but seems to be present in smaller specialised muscles (e.g. the eye) (Toniolo et al., 2005). Apart from the structural and metabolic differences between the three fibre types, maximum force and power output capacities increases from type I, IIA to IIX fibres (Bottinelli, 2001; Kohn and Noakes, 2013).

Studies on skeletal muscle from humans and animals active in various sporting disciplines (i.e. exercise trained *vs.* sedentary; resistance *vs.* endurance trained), have confirmed that fibre type and their diameters, as well as marker enzyme activities of the various metabolic pathways, were good indicators of muscle power and flux capacity through the different metabolic pathways, respectively (Bottinelli, 2001; Gollnick et al., 1972; Pette, 1985; Rivero et al., 2007). In man, it is well known that heavy resistance training increases muscle fibre size, shifts fibres towards predominantly type IIA fibres and increases glycolytic capacity (Tesch et al., 1989). Muscle from endurance trained individuals predominantly present with type I muscle fibres and high oxidative capacities (high mitochondrial content within fibres) for ATP to be derived from oxidation of fat and carbohydrates (Essén-Gustavsson and Henriksson, 1984; Kohn et al., 2007b).

Our group has investigated the skeletal muscle characteristics from a variety of wild animal species, focussing primarily on the morphology, fibre type, metabolism and contractility of the *Vastus lateralis* muscles to better understand muscle function (Curry et al., 2012; Kohn and Noakes, 2013; Kohn et al., 2011b; Kohn et al., 2011a). In conjunction with research on other species, it has now become evident that the felids (lion, tiger, cheetah and caracal) possess muscles that have predominantly type IIX muscle fibres, and relies primarily on anaerobic pathways to generate ATP for muscle contraction (Hyatt et al., 2010; Kohn et al., 2011a; Williams et al., 1997). Additionally, the lack of abundant mitochondria and poor oxidative enzyme capacity within their muscles confirmed that felids are sprinters and lack the capacity to withstand fatigue. On the other hand, muscles from their prey (e.g. wildebeest and various antelope species) also contain a large proportion of pure type IIX fibres, but metabolically, their muscles are highly oxidative and glycolytic, thus giving these animals the advantage of added endurance to escape predation (Curry et al., 2012; Kohn et al., 2011b; Kohn et al., 2007c).

Importantly, differences do exist between the various animal species. It appears that the heavier an animal, the less type IIX fibres are present. Furthermore, a strong positive relationship was shown between the percentage of type IIX fibres and the reported maximum sprinting capability of the wild animals (Curry et al., 2012). As a continuation to better understand skeletal muscle, we present data on three antelope species, each indigenous to southern Africa, namely the mountain reedbuck (*Redunca fulvorufula* – Afzelius 1815), blesbok (*Damaliscus pygargus phillipsi* – Pallas 1767) and greater kudu (*Tragelaphus strepsiceros* – Pallas 1766).

The mountain reedbuck is the smallest of the three antelope, with adult males weighing approximately 30 kg and the females 28 kg (Skinner and Chimimba, 2005). Only the males have horns. Its natural habitat is in mountainous regions, located on the eastern parts of South Africa, south-eastern parts of Botswana, and isolated pockets on the African continent, where herds range between 3 to 8. They have even been spotted on Kilimanjaro (Tanzania) and the Drakensberg (South Africa) at altitudes of 4 560 meters and 2 200 meters above sea level, respectively. These animals are apparently not as fast as other antelopes (e.g. springbok), but the exact maximum speed has not been measured. However, the author has witnessed first hand their ability to swiftly run up hills at gradients of approximately 30 degrees.

The weight of adult male and female blesbok ranges between 56 and 61 kg (Skinner and Chimimba, 2005). It is difficult to distinguish between sexes as both have horns. They roam the flatter grasslands of the eastern, central and northern parts of South Africa and tolerate heat very well. Relying on their speed to escape predation, blesbok are fast runners (anecdotal evidence suggests they can potentially reach a speed of approximately 70 km·h^−1^), but poor at jumping.

Lastly, the greater kudu is one of the heaviest antelopes, with the males distinctly larger than females (average male: 300 kg; average female: 150 kg). Kudu males are renowned for having the longest horns in the animal kingdom, spanning almost 1.5 meters in length. Their habitat includes the dryer parts of southern Africa (South Africa, Namibia, Botswana, Zimbabwe and the western part of Mozambique) and eastern parts of Africa (Skinner and Chimimba, 2005). On a performance level, greater kudu are considered the slowest runners of all antelope, but skilled jumpers, clearing a 2-meter high fence with the greatest of ease.

The aim of this study therefore was to investigate the muscle fibre type, morphology and metabolism of these three antelope species and elucidate whether their muscle parameters complement their physical performance attributes. It is hypothesised that the content of type IIX fibres will significantly decrease as the weight of the animal increases. Furthermore, the metabolic profile in the skeletal muscle of these three species will follow the same pattern (i.e. high oxidative and glycolytic capacities) as that observed in other studies on antelopes.

## RESULTS

### Animals and grouping

The three animal groups were heterogeneous with regards to sex, but all adult. All the blesbok were female, whereas the 4 kudus were male. Of the 13 mountain reedbuck sampled, 11 were female. Although statistics was not feasible, closer inspection of the data showed that the two males did not affect the overall mean of the muscle parameters, and being well within the ranges of the females. Therefore, the data for mountain reedbuck were grouped.

### MHC migration

**Fig. 1.**
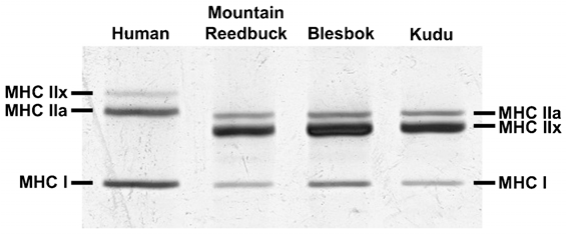
Myosin heavy chain isoforms from human, mountain reedbuck, blesbok and kudu *Vastus lateralis* muscles separated by SDS-PAGE.

Fig. 1 shows the migration profiles of the MHC isoforms from human and the three antelope species. Three bands could be identified for human, with the top band corresponding to MHC IIx, the middle to MHC IIa and the bottom band, MHC I. According to previous studies using a different set of monoclonal antibodies (A4.74 specific to MHC IIa and N2.261 specific to MHC I and IIa), the bands for the kudu and blesbok correspond to (from top to bottom) MHC IIa, MHC IIx and MHC I (Kohn et al., 2007c). No additional bands was identified that could indicate the presence of the MHC IIb isoform.

### Specificity of the MHC antibodies used for IHC

**Fig. 2.**
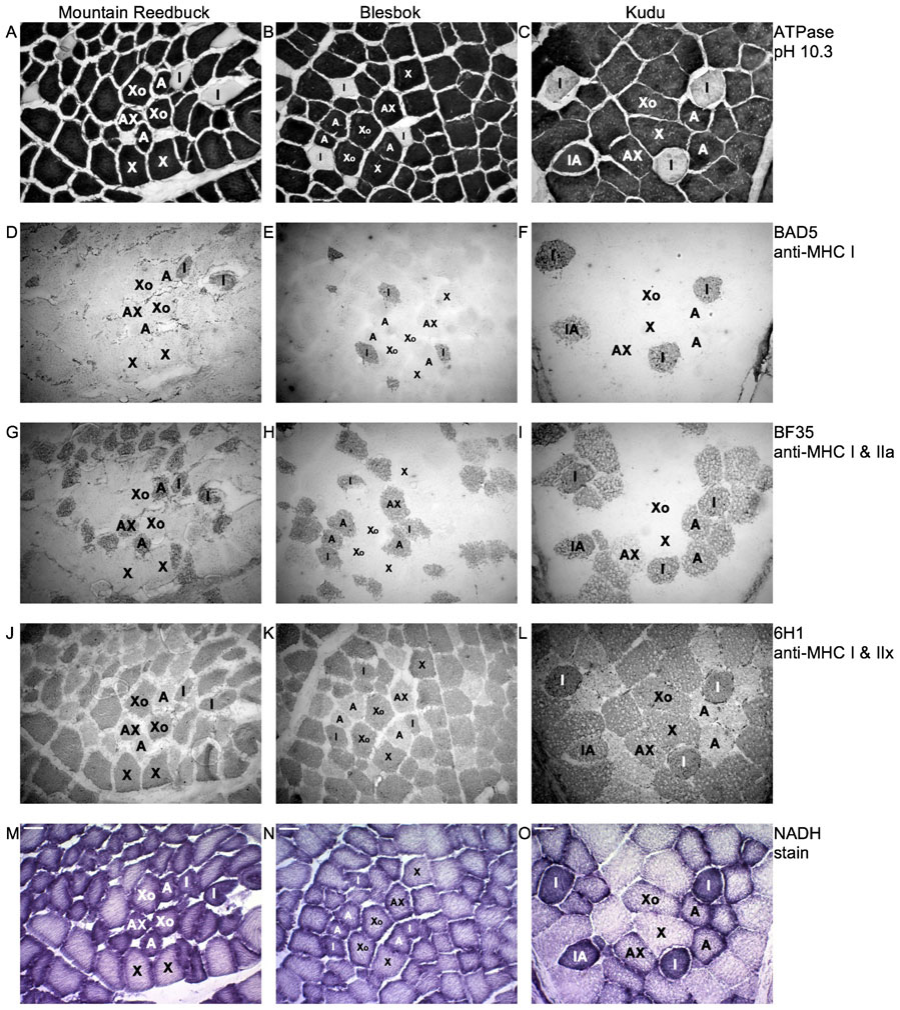
Histology of mountain reedbuck, blesbok and kudu *Vastus lateralis* muscles. Fibres were classified as types I (I), IIA (A), IIAX (AX) and IIX (X). Type IIX fibres presenting with high oxidative capacity are labelled as Xo. (A–C) ATPase stain at pH 10.3. Type I fibres are clear. All other fibres are stained dark. Immunohistochemistry using an antibody specific to: (D–F) MHC I (BAD5); (G–I) MHC I and IIa (BF35); (J–L) MHC IIx (6H1). Note cross-reactivity with type I muscle fibres in all three species. (M–O) NADH stain showing oxidative capacity of muscle fibres.

The NADH-tetrazolium stain, ATPase stain at pH 10.3 and immunohistochemistry using antibodies specific to MHC I (BAD5), MHC I and MHC IIa (BF35), and MHC IIx (6H1) are presented in Fig. 2. BAD5 (Fig. 2D–F) only reacted with type I fibres and corresponded to pure type I fibres, corresponding to the type I profiles depicted in the ATPase stains (Fig. 2A–C). Fibres containing either pure MHC I or MHC IIa, both, or MHC IIa in combination with MHC IIx reacted with BF35 (Fig. 2G–I). The monoclonal 6H1 reacted with fibres containing MHC IIx, but also cross-reacted with pure type I fibres (Fig. 2J–L). Fig. 2M–O depicts the oxidative capacity of the different fibre types. Fibres containing MHC I primarily had darker staining intensities compared to fibres containing MHC IIa or IIx, indicating a greater oxidative capacity. The MHC IIb isoform could not be detected in these slides using the specific MHC IIb antibodies (BF-F3 and 10F5) (data not shown).

### Muscle fibre type and size

The relative fibre type (MHC isoform content) and actual fibre type determined from immunohistochemistry are presented in Tables 1 and 2, respectively. These two independent measures of fibre type complemented each other very well. Accordingly, all three animal groups had very little type I muscle fibres (6 to 14%), whereas the majority of the fibres belonged to the pure type IIX group. Blesbok had significantly less type I fibres than kudu. In all animal species, the MHC IIa and type IIA fibre content ranged between 21–36% and 18–34%, respectively. The two larger species had significantly more type IIA fibres than mountain reedbuck and was echoed by their respective MHC isoform content. The content of the MHC IIx isoform in mountain reedbuck was significantly more than in blesbok and kudu, but significance was only found between kudu and mountain reedbuck using immunohistochemistry (Table 2). Although the type IA hybrid fibres are shown in the kudu immunohistochemical sections, very few were present and considered negligible.

**Table 1.**
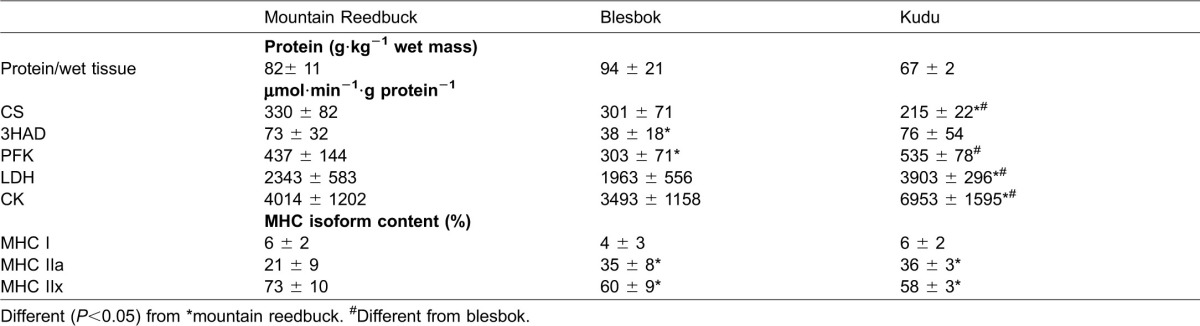
Enzyme activities and myosin heavy chain (MHC) isoform content of the Vastus lateralis from three antelope species

**Table 2.**
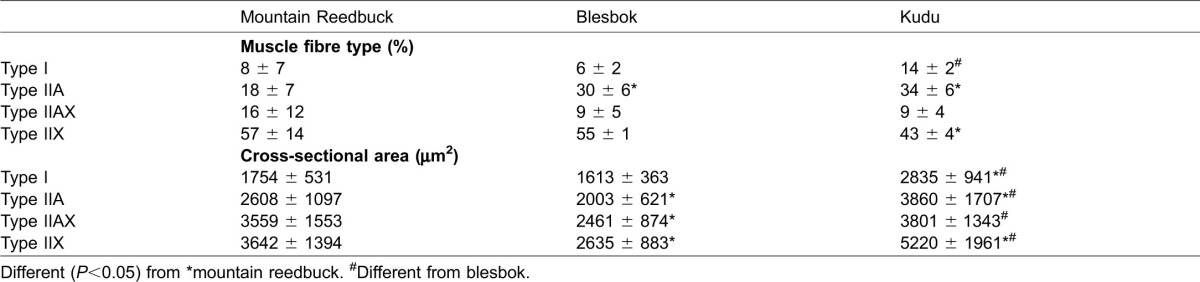
Muscle fibre type, cross-sectional area (CSA), and correlations between oxidative capacity of fibres and their CSA of the *Vastus lateralis* from three antelope species

The cross-sectional areas (CSAs) of each fibre type from the three species are depicted in Table 2. In all three species, type I fibres were the smallest, with a steady increase in size as the fibre types increased in reported contractile speed from type IIA, IIAX and IIX. However, there was a marked species effect (*P*<0.001). The mean CSA of each species amounted to 3324±1447 µm^2^, 2372±858 µm^2^ and 4323±1920 µm^2^ for mountain reedbuck, blesbok and kudu, respectively.

### Muscle metabolism

Representative markers of the metabolic pathways are presented in Table 1. The oxidative capacity (CS activity) was the highest in the mountain reedbuck and blesbok, but their capacity to produce lactate, lower than kudu (*P*<0.05). PFK activities were different between all three species, with the highest levels recorded in kudu, and the lowest in blesbok. The capacity of β-oxidation (represented by 3HAD) was 2-fold greater in mountain reedbuck and kudu, compared to blesbok. Finally, kudu muscle had the greatest capacity in CK activity.

### Oxidative capacity of fibre types

**Fig. 3.**
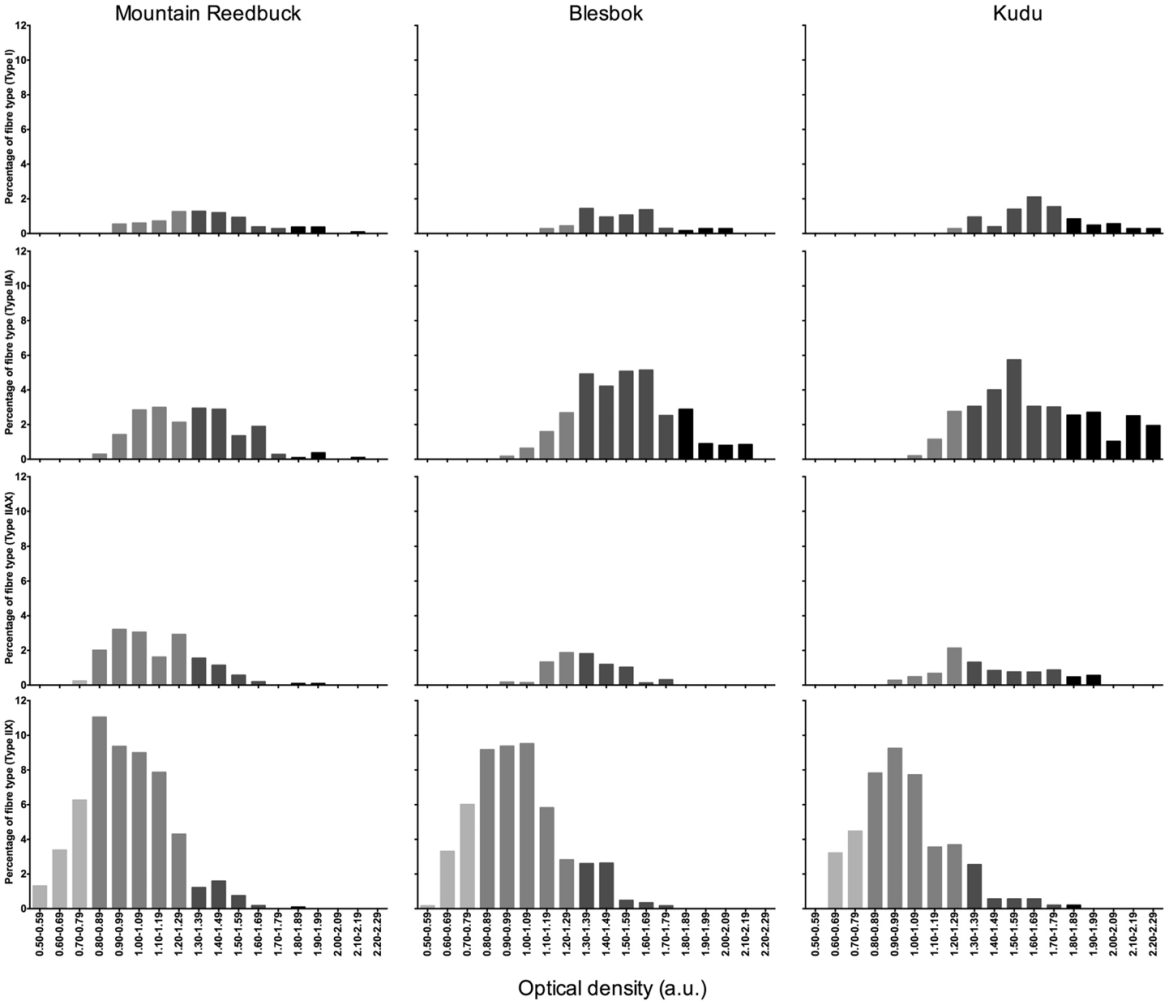
Frequency distribution plots (expressed as a percentage of the total number of fibres) of the oxidative capacity (optical density (OD)) from mountain reedbuck, blesbok and kudu *Vastus lateralis* muscle. Each bar was calculated as the number of fibres that obtained OD values in a specific range (e.g. from 0.70 to 0.79 OD).

The oxidative capacity of each muscle fibre was quantified by determining the optical densities (OD) from the NADH stains (Fig. 2M–O) and the frequencies presented graphically (Fig. 3). The highest oxidative capacities were recorded in the type I and IIA fibres, followed by the type IIAX and type IIX fibres. The presence of oxidative type IIX fibres was also found in all three species (labelled Xo, Fig. 2M–O). There was a wide variation in oxidative capacities between and within the individual fibre types from the three species. The range was especially large for type IIA and type IIX fibres. Mountain reedbuck did seem to have an overall lower oxidative capacity compared to that from kudu and blesbok, but this was not statistically significant.

### Correlations

No relationships were found between fibre type and metabolism, or metabolism and oxidative capacity of the fibre types for any of the species, or combined. However, strong negative relationships were observed between CSA and oxidative capacity of the fibres (determined from Fig. 3 and Table 2) for the three species (Spearman's r: mountain reedbuck: −0.57, *P*<0.0001; blesbok: −0.45, *P*<0.0001; kudu: −0.38, *P*<0.0001). When the fibres were divided into their respective fibre types, only fibres from mountain reedbuck showed a continuation of this strong relationship (Spearman's r: type I fibres: −0.47, *P*<0.0001; type IIA fibres: −0.68, *P*<0.0001; type IIAX: −0.40, *P*<0.0001; type IIX fibres: −0.36; *P*<0.0001), whereas only the type IIX fibres from blesbok were significant (Spearman's r: −0.21, *P*<0.0001). The kudu had poor to no relationships for CSA and fibre oxidative capacity.

## DISCUSSION

This is the first study to perform a comprehensive analysis of skeletal muscle from mountain reedbuck, blesbok and kudu. In comparison to previous studies on muscle from human, felid and other ungulates (see text below on muscle and fibre metabolism), all three antelope species had a high oxidative metabolic capacity, high mitochondrial content, as well as a high glycolytic capacity. Also, all three species had predominantly type IIX muscle fibres, followed by type IIA, and very few type I fibres.

### Muscle fibre type and size

Muscle fibre type of the three species was determined using antibodies specific to the three MHC isoforms, as well as by separating the MHC isoforms to obtain the relative fibre type profile. All three species expressed the MHC I, IIa and IIx isoforms as separated using SDS-PAGE. The order of migration conformed to that from previous studies using either kudu, blesbok, horse or wildebeest muscle (Curry et al., 2012; Kohn et al., 2011b; Kohn et al., 2007c; Rivero et al., 1999). As has been observed in most studies on humans and animals (e.g. lions, caracal, horses and antelopes), there seems to be consensus that the MHC I and IIa isoforms are similar in size between these species. However, certain species seem to have a different size for their respective MHC IIx isoform. Specifically, the human MHC IIx isoform is larger than its MHC IIa (as seen in Fig. 1), whereas in the antelopes, it is slightly smaller than their respective MHC IIa. In felids, including the lion and caracal, the MHC IIx isoform seems even smaller than that from antelopes (Kohn et al., 2011a). How these size differences contribute to overall muscle function is still unclear.

It is well known that the fibre types differ in their respective contractile properties and metabolism (Bottinelli, 2001; Pette, 1985). In comparison, human *Vastus lateralis* contains vast numbers of type I and IIA fibres, even in an untrained state (Gollnick et al., 1972; Harber and Trappe, 2008; Klitgaard et al., 1990; Kohn et al., 2007a). To date, no study has shown a predominance of type IIX fibres in large muscle groups from humans. In contrast, most of the large wild animals studied thus far show a predominance of type IIX muscle fibres. The felids (lion, cheetah, caracal and tiger) had a predominance of type IIX fibres, ranging between 50 to 70% in their respective *Vastus lataralis* muscles (Hyatt et al., 2010; Kohn et al., 2011a). Similarly, prey of these animals (e.g. springbok, black wildebeest and fallow deer) and the llama have also been shown to contain large numbers of type IIA and IIX fibres, correlating well to their maximum sprinting ability (Curry et al., 2012; Graziotti et al., 2001; Kohn et al., 2011b; Spurway et al., 1996). In accordance, the three species studied here all had a high proportion of type IIX fibres. Particularly, mountain reedbuck had very few type I and IIA fibres, whereas the blesbok and kudu had more type IIA fibres than mountain reedbuck (confirmed by their respective MHC IIa content) (Tables 1 and 2). The mountain reedbuck had a similar muscle fibre type profile as that from springbok (MHC IIx: 73±10% vs. 68±7%; MHC IIa: 21±9% vs. 27±5%, respectively) (Curry et al., 2012). Both these species weigh approximately 30 kg. Heavier antelope, like the blesbok and the fallow deer (approximately 45–55 kg), also had similar profiles to one another (MHC IIx: 55±1% vs. 60±9%; MHC IIa: 35±8% vs. 38±7%) (Curry et al., 2012). With these associations, it was anticipated that the fibre type from the *Vastus lateralis* of the much heavier kudu (∼300 kg) would more closely resemble that of heavier ungulates, such as the black wildebeest. This hypothesis was, however, not true as the kudu in the present study had more MHC IIx than black wildebeest (58±3% vs. 32±9%, respectively) (Kohn et al., 2011b). It may therefore indicate muscle specialisation of the kudu to make them excellent at jumping, whereas the predominance of type IIA fibres in black wildebeest allows for endurance capacity (i.e. migration). Whether other species within this family shows similar muscle profiles is still to be determined.

Although other factors may influence the contractile properties of muscle fibres (e.g. variation in the myosin light chain isoform expression), the major contributor to the force output of a fibre is its size (Bottinelli, 2001). Additionally, strength training in humans increases fibre CSA, resulting in increased absolute force (Cristea et al., 2008; Shoepe et al., 2003). Generally, type IIX fibres are larger than the type IIA fibres, and they, in turn, larger than type I fibres. Human fibres are generally larger in size than those from animals (Curry et al., 2012; Karlström et al., 1994; Kohn et al., 2011b; Kohn et al., 2011a). It is still unclear why certain species have smaller fibres and may partly be attributed to genetics. Recently, the relationship between fibre size and their respective oxidative capacities were investigated to elucidate why oxidative fibres are normally smaller than their glycolytic counterparts (van Wessel et al., 2010). In humans, that is, the hypothesis seems to revolve around the type of exercise (resistance *vs.* endurance) performed, which results in altered signalling cascades that would either activate cellular hypertrophy or mitochondrial biogenesis, or both. The average fibre CSA between the three species in the present study differed by 1.4× between blesbok and mountain reedbuck, 1.3× between kudu and blesbok and 1.8× between blesbok and kudu. The CSAs also correlated well with fibre oxidative intensities (Spearman's r: mountain reedbuck: −0.57; blesbok: −0.45; kudu: −0.38), revealing *P* values <0.0001 and thus agreed with van Wessel et al. (van Wessel et al., 2010). However, once the fibres were divided into their respective fibre types, two species lost their respective strong relationships between oxidative capacity and CSA. Thus, it seems that a potential genetic component other than fibre type or training status plays a greater contributing factor towards muscle fibre CSA and metabolism of the wild animals. It needs to be highlighted that the animals used in this study were all wild and not captive, therefore the assumption is that they are not detrained, as would be the case for zoo animals.

### Muscle and fibre metabolism

The two most prominent metabolic characteristics observed in the muscle of the three species were their high i) oxidative and ii) glycolytic capacities. Fibres had a wide range in oxidative capacity as indicated by the different intensities from the NADH stain in Fig. 2M–O. Darker fibres are usually associated with being more oxidative and hence, containing more mitochondria than lighter fibres (Novikoff et al., 1961). The oxidative capacity of the fibres was not strongly associated with fibre type – the latter showing large overlap in oxidative capacities (Fig. 3). This is in agreement with previous studies using wild ungulates that included reindeer, black wildebeest, springbok, fallow deer, dik dik, topi, waterbuck, impala and hartebeest (Curry et al., 2012; Essén-Gustavsson and Rehbinder, 1985; Hoppeler et al., 1981; Kohn et al., 2011b; Kohn et al., 2005; Spurway et al., 1996). The oxidative capacities are also comparable to that observed in endurance trained humans (Chi et al., 1983; Essén-Gustavsson and Henriksson, 1984; Kohn et al., 2007b; Kohn et al., 2011c). Wild felids, including lion, caracal and cheetah, have very poor oxidative capacities reflected by their respective low CS and 3HAD activities, and poor NADH staining intensities (Kohn et al., 2011a; Williams et al., 1997). Additionally, the type IIX fibres from ungulates seem to possess another characteristic unique to these types of species, namely oxidative type IIX fibres. This is in agreement with previous studies using black wildebeest, springbok, reindeer and fallow deer (Curry et al., 2012; Essén-Gustavsson and Rehbinder, 1985; Kohn et al., 2011b). With regards to anaerobic capacities (LDH and CK), all the wild animals seem to share an equally high capacity to metabolise glucose and glycogen, whereas that from humans are almost 4 fold lower (Kohn et al., 2011a).

### What about their running ability and endurance capacity?

An important body of research currently lacking is sound scientific evidence for performance (running, jumping, endurance ability) of wild species. Thus, the majority of points discussed here would be merely speculation, but seems to confirm some anecdotal aspects of the performance ability for the three species studied here (Garland, 1983).

Mountain reedbuck is a small antelope living on the slopes of medium sized mountains. They do have the ability to run faster than the average antelope. They also have the ability to scale these mountains at great speeds. They therefore would require muscle with properties that produce explosive power (e.g. IIX fibres) and a mechanism that can adequately produce ATP at a high rate. Although not the highest values, the muscle from mountain reedbuck seems to harbour all these characteristics (Tables 1 and 2). Their small body size and weight may also be beneficial in their running ability, resulting in a better power to weight ratio.

Kudu, on the other hand, is considered the largest antelope, but also the slowest of all. In comparison to the two species presented here and to other species previously published, their CS activities seem the lowest, but comparable 3HAD activities, the latter indicating similar beta-oxidation capacities (Curry et al., 2012; Kohn et al., 2011b). Both PFK and LDH activities were very high, and, compared to the other species, their CK values were also the highest. Black wildebeest, known to be a migrator, weighing approximately 160 kg and can achieve a running speed of 70 km·h^−1^, only contained approximately 30–40% type IIX fibres in their *Vastus lateralis* (Kohn et al., 2011b). Kudu had, on average, 60% type IIX fibres, and considering the CSA of their type IIX fibres, some of the largest fibres of the three species investigated here. However, it is highly unlikely that a kudu would reach such great speeds (e.g. above 50 km·h^−1^), most likely because of its anatomical build and very long neck that would greatly influence its running biomechanics. Nevertheless, kudu is well known for their jumping ability, clearing a 2 meter high fence from a stationary position with the greatest of ease (Skinner and Chimimba, 2005). Thus, the high type IIX fibre content, large CSA and very high CK activities adequately explain this unique jumping ability.

Although Garland reported maximum speeds for various animal species (measured and anecdotal), the blesbok was not among those (Garland, 1983). Additionally, it does not seem that body weight is a good predictor of running speed. However, from their muscle characteristics (high proportion of type IIX fibres) and the previously strong correlation between type IIX fibre content and running speed (r = 0.80, *P*<0.001) reported by Curry et al. (Curry et al., 2012), it would seem that they could achieve speeds of up to 70 km/h. Although these animals are almost twice the size of the mountain reedbuck, their fibre CSAs were significantly smaller than mountain reedbuck and kudu. In fact, their fibres seem to be the smallest when compared to black wildebeest, springbok and fallow deer (Curry et al., 2012; Kohn et al., 2011b). The blesbok's anaerobic metabolic capacity to use glucose and glycogen as fuel (PFK and LDH), as well as rapid ATP replenishment from phosphocreatine stores, was the lowest of the three species. Speculatively, the lack in the ability for rapid ATP production, as well as having small fibres could partly explain why they are considered as being extremely poor at jumping (Skinner and Chimimba, 2005).

The present study was aimed at comparing the muscle morphology to that from humans. In humans, the *Vastus lateralis* is most commonly sampled. Very little data is available from other animal species using this particular muscle group. Nevertheless, steers are known to be poor at running and weigh between 300 and 500 kg, depending on the breed, whereas horses are fast and have immense endurance capacity. Both animals expressed primarily type I (∼37%) and IIA fibres (∼53%), with less than 10% type IIX fibres in their *Vastus lateralis* (Karlström et al., 1994). Overall, horses had higher oxidative but lower glycolytic capacity than the steers, explaining their better resistance to fatigue. Horses also had larger muscle fibres, which could also explain their excellent jumping capability. Another animal that is not considered a good runner is the camel (*Camelus dromedaries*) (Cluer et al., 1994). Being able to reach a maximum speed of approximately 40 km/h, their *Vastus lateralis* primarily comprises type IIA fibres (∼50%) and equal amounts of type I and type IIA fibres. They derive their ATP predominantly from fat metabolism having high oxidative (CS and 3HAD activities), but low glycolytic (LDH) capacities. In comparison, the antelopes are predominantly expressing type IIX fibres, and have both high oxidative and glycolytic capacities. Their fibres have a wide range in oxidative capacities that is not restricted to a particular fibre type. These muscle parameters, therefore, are partly able to explain the physical performance characteristics of the three species investigated.

## MATERIALS AND METHODS

### Animals and tissue sampling

Professional hunters randomly shot antelope on game farms during the annual cropping season in the Pearston–Somerset-East area located in the Eastern Cape province of South Africa. Muscle samples from the *Vastus lateralis* were collected post mortem (within 4 hours of death) from 13 female blesbok, 2 male and 11 female mountain reedbuck and 4 male greater kudu. The sampling site where the muscle was obtained from was determined as half the distance between the knee and hip joint. Depending on the size of the animal, the depth was approximately one third the thickness of the quadriceps. An incision was made through the hide and a block of tissue (±1 cm^3^) removed, divided into smaller parts, rapidly frozen in liquid nitrogen and stored in a cryo-preservation tank (−200°C). Samples were transported to the laboratory in liquid nitrogen and stored at −87°C until analyses.

### Homogenisation of tissue

Samples were prepared for enzyme analyses as described (Curry et al., 2012). For every 1 mg tissue, 19 µl of 100 mM potassium phosphate buffer, pH 7.30, was added (1:19), homogenised on ice and subsequently sonicated (Virtis Virsonic Ultrasonic Cell Disrupter 100). The protein concentration of each sample was determined using the method described (Bradford, 1976). Enzyme assays were performed using the homogenate, whereas a small amount of homogenate was diluted with sample buffer (5% β-MEtOH, 2.5% SDS, 10% glycerol, 62.5 mM Tris, pH 6.8 and 0.1% bromophenol blue). The latter samples were heated to 95°C for 3 minutes and used to determine the MHC isoform content.

### Enzyme analyses

Enzyme activities, serving as markers for the respective metabolic pathways, were: CK (EC 2.7.3.2) for rapid ATP replenishment capacity, PFK (EC 2.7.1.11) for flux capacity of glycolysis, LDH (EC 1.1.1.27) for lactate production, CS (EC 2.3.3.1) for Krebs cycle oxidative capacity and 3HAD (EC 1.1.1.35) for fat oxidation capacity. Enzyme activities were determined spectrophotometrically at 25°C using modified methods described (Kohn et al., 2011a). The final volume for CS, 3HAD, CK and PFK was reduced to 0.5 ml, whereas that for LDH was kept at 1 ml. Each enzyme assay was performed over a period of 5 minutes, the slope determined and expressed relative to the amount of tissue (µmol·min^−1^·g protein^−1^).

### Myosin heavy chain isoform content

The relative MHC isoform content of the *Vastus lateralis* from the three species was determined using SDS-PAGE as described (Kohn et al., 2007c). A human *Vastus lateralis* sample (obtained from a previous study for which ethical approval was obtained) containing all three MHC isoforms, was included to serve as marker of isoform migration (Kohn et al., 2011a). Gels were run in the cold for 24 hours, first at 70 V constant for 4 hours, followed by 20 hours at 275 V, and subsequently silver stained, scanned and analysed using Un-Scan-It (Silk Scientific Corporation). Three isoforms for each species were identified according to their migration on the gel. The relative content of each band was expressed as a percentage of the total pixel density of all three bands.

### Histology and immunohistochemistry

Serial cross-sections (10 µm) were cut from frozen muscle in a cryostat set to −25°C and stained for ATPase activity (pH 10.3) to aid in fibre boundary identification during subsequent immunohistochemical stains and analyses (Brooke and Kaiser, 1970). The oxidative capacities of the fibres were achieved by staining the mitochondria with an NADH-nitro blue tetrazolium reaction for 30 minutes at 37°C (Curry et al., 2012; Novikoff et al., 1961).

For immunohistochemistry, three monoclonal antibodies against the MHC isoforms were used – BAD5 recognises exclusively MHC I, BF35 MHC I and IIa, and 6H1 MHC IIx (Lucas et al., 2000; Schiaffino et al., 1989) – and visualised using a DAB staining kit (DAKO, Denmark). Sections were viewed under a light microscope at 10× magnification, photographed (AxioVision, Zeiss, Germany) and fibres typed according to their reactivity to the specific antibodies. These were identified as either type I, IIA, IIAX or IIX. Once identified, the CSA of the fibres was determined using the ImageJ for Mac software package (Rasband, W.S., ImageJ, U. S. National Institutes of Health, Bethesda, Maryland, USA, http://imagej.nih.gov/ij, 1997–2011).

The oxidative capacity of each fibre type was determined by analysing the OD of each fibre type using pre-calibrated software (ImageJ), whereafter frequency distribution curves (in percentage) were generated for each fibre type and species (as described in detail by Curry et al., 2012). Briefly, the number of fibres in a specific range of OD values (e.g. the number of fibres between 0.70–0.79 OD expressed as a percentage of the total number of fibres) were counted and expressed as a percentage of the total number of fibres.

### Statistical analyses

Values are expressed as means ± standard deviation. Inter-species differences were determined using the non-parametric Kruskal–Wallis ANOVA with a Dunn's *post hoc* test. Relationships were determined using the Spearman correlation coefficient for non-parametric data. For all statistical analyses, significance was set at *P*<0.05. However, the significance was increased to a *P*<0.01 for relationships between fibre oxidative capacities and CSAs. Clarification: uppercase letters are used for fibre types derived from histology, whereas lowercase letters refer to fibre types derived from the MHC isoform analyses using SDS-PAGE.

### List of abbreviations

3HAD: 3-hydroxyacyl co A dehydrogenase; CK: creatine kinase; CS: citrate synthase; CSA: cross-sectional area; LDH; lactate dehydrogenase; MHC: myosin heavy chain; OD: optical density; PFK: phosphofructokinase; SDS-PAGE: sodium dodecyl sulphate polyacrylamide gel electrophoresis.
